# Case Report: Effect of preventive intravenous iron infusion on postoperative hemoglobin levels in a female patient with latent iron deficiency anemia before total hip arthroplasty

**DOI:** 10.3389/fmed.2025.1586760

**Published:** 2025-10-22

**Authors:** Nazerke Zhanarystan, Shamil Shayakhanov, Maiya Konkayeva, Assiya Kadralinova, Aidos Konkayev

**Affiliations:** ^1^Department of Anesthesiology and Intensive Care, Astana Medical University, Astana, Kazakhstan; ^2^Department of Anesthesiology and Intensive Care, National Scientific Center of Traumatology and Orthopedics Named after Academician N.D. Batpenov, Astana, Kazakhstan; ^3^Department of Infectious Diseases and Clinical Epidemiology, Astana Medical University, Astana, Kazakhstan

**Keywords:** preoperative hemocorrection, preoperative latent anaemia, Orthopedics, iron deficiency, total hip replacement, case report

## Abstract

This article presents a case of orthopedic treatment of a 51-year-old patient with latent iron deficiency anaemia and congenital dysplasia of both hip joints. The patient was admitted with complaints of severe pain and restriction of movement in the right hip joint, impaired joint function, shortening of the right lower limb, and lameness. A year prior, the patient had undergone surgery on the left hip joint to treat the same complaints. During the left hip surgery, the patient required haemotransfusion in the postoperative period to manage severe posthemorrhagic anaemia. In this clinical case, under equal conditions, the patient underwent preoperative preparation and administration of ferric carboxymaltose to compare the efficacy of iron deficiency correction versus no correction. No haemotransfusion was required in the postoperative period. The uniqueness of this clinical case is represented by the fact that the example of one patient showed the importance of preparation for orthopedic intervention and iron deficiency correction to avoid haemotransfusions, which are closely associated with infectious complications. In conclusion, preoperative correction of iron deficiency and treatment of latent anaemia in this clinical case was associated with no need for haemotransfusion in the postoperative period compared to no preoperative preparation. Further studies are needed.

## Introduction

A significant percentage of individuals who need surgery each year are found to have preoperative anaemia. Preoperative anaemia is present in about 39.1% of surgical patients on average (1, 2).

The main cause of anaemia, iron deficiency, continues to be a major global health burden, affecting a large number of people worldwide. Globally, the number of iron deficiency cases is increasing yearly and is expected to reach 1439.99 million by 2050, representing about 18% of the world’s population (3). Approximately 36% of people worldwide suffer from latent anaemia associated with iron deficiency, according to WHO data currently available (4). Therefore, the number of patients with latent iron deficiency anaemia may be significantly higher worldwide at present due to the prevalence of chronic gastritis and other GI diseases linked to inadequate dietary intake.

Preoperative anaemia is a prevalent condition that significantly increases the likelihood of an adverse postoperative outcome. Implementing a patient blood management concept is essential to reducing the negative postoperative occurrences linked to anaemia.

The presented case is unique because the patient had underlying anemia with low iron levels before a major orthopedic intervention with anticipated volume blood loss. Latent iron deficiency was probably caused by low absorption due to the patient’s chronic gastritis. With relatively equal conditions and the same volume of surgical intervention in this clinical case, we used an intravenous infusion of ferric carboxymaltose in the preoperative preparation, which probably influenced the more successful outcome of the operation.

## Case description

### Patient information

**Figure 1:**
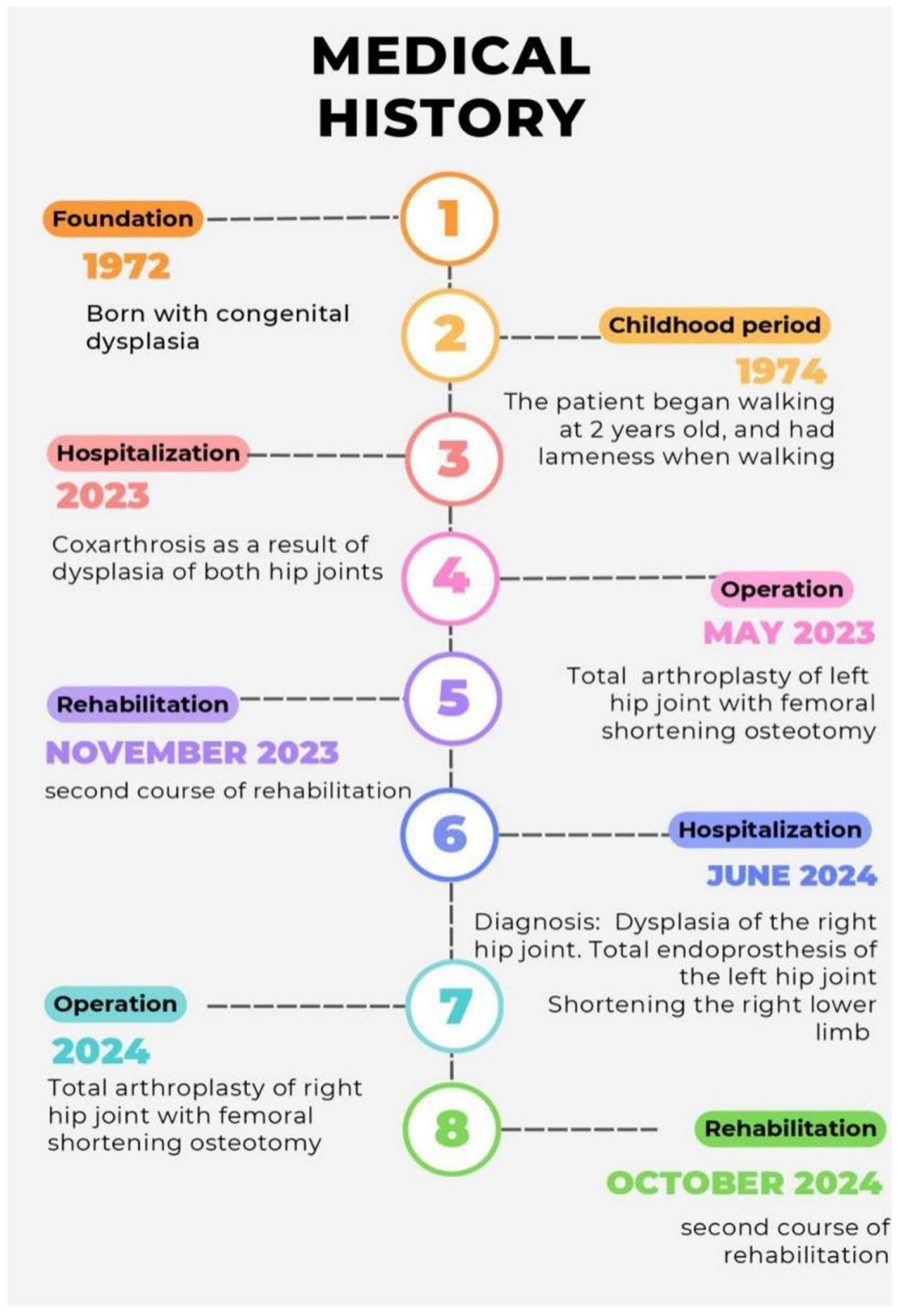
The medical history.

A 51-year-old female patient was admitted for a planned unilateral hip arthroplasty with complaints of severe pain and restriction of movement in the right hip joint, joint dysfunction, and lameness. She believes that she has been ill since childhood when the pain syndrome first appeared. After examination, she was diagnosed with “congenital dysplasia.” Consequently, she received repeated inpatient conservative treatment for dysplastic coxarthrosis, the timeline is presented in Figure 1. Due to the ineffectiveness of non-surgical therapy and increasing pain syndrome, the first surgery was performed in May 2023 on the left hip joint in the scope of total endoprosthesis of the left hip joint with Zimmer Wagner implant with shortening osteotomy. In the postoperative period, hemoglobin decreased to 70 g/L, which required haemotransfusion of allogeneic blood. This hospitalization was due to the necessity of surgical treatment of the right hip joint in the amount of total hip replacement of the right hip joint with shortening osteotomy. The patient’s concurrent conditions include chronic bronchitis, arterial hypertension, chronic gastroduodenitis, and iron deficiency anemia, for which she occasionally takes oral iron supplements. Gynecologic anamnesis and heredity are unremarkable. However, she has an allergic reaction to metoclopramide in the form of anaphylactic shock.

### Clinical findings

The patient’s objective status was not different from normal. Her weight was 62 kg with a height of 158 cm; the ideal body weight according to Broca’s formula was 52.3 kg. Her body mass index was 24.8 kg/m^2^. The skin and visible mucous membranes were physiologically colored, without rash and peripheral edema. Respiration was independent with a respiratory rate of 17 breaths per minute. Hemodynamic parameters were stable, at the time of examination blood pressure was normotonic - 117/75 mm Hg and heart rate - 75 beats per minute. The genitourinary and digestive systems’ organs are without any abnormalities. On examination of the musculoskeletal system, the patient moves independently, with padding on the right shoe 4 cm high, and limping on the right lower limb. Movements in the right hip joint are painful and limited: left hip joint flexion-flexion 90°-0–180° abduction-adduction 30°-0° -20° rotation from outside to inside 20°-0–15°. Movement in the left hip joint is painless. The postoperative scar of the left hip joint is without features. There are no vascular and neurological disorders in the periphery of the lower extremities.

### Timeline

The chronology of patient’s medical history from the time of admission to the time of discharge from hospital is highlighted in Figure 1.

### Diagnostic assessment

The results of laboratory tests on admission revealed latent iron deficiency anaemia, with an erythrocyte count of 4 × 10^12^/L and hemoglobin of 132 g/L. The patient underwent additional examination for iron deficiency before surgery because history of treatment for iron deficiency anaemia. On admission in the biochemical blood test, iron was 6.8 mmol/L, transferrin 216 mg/dL, ferritin 92.8 mcg/L, at discharge iron was 4.3 mmol/L; transferrin 138.0 mg/dL; ferritin level 306.3 mcg/L.

**Figure 2:**
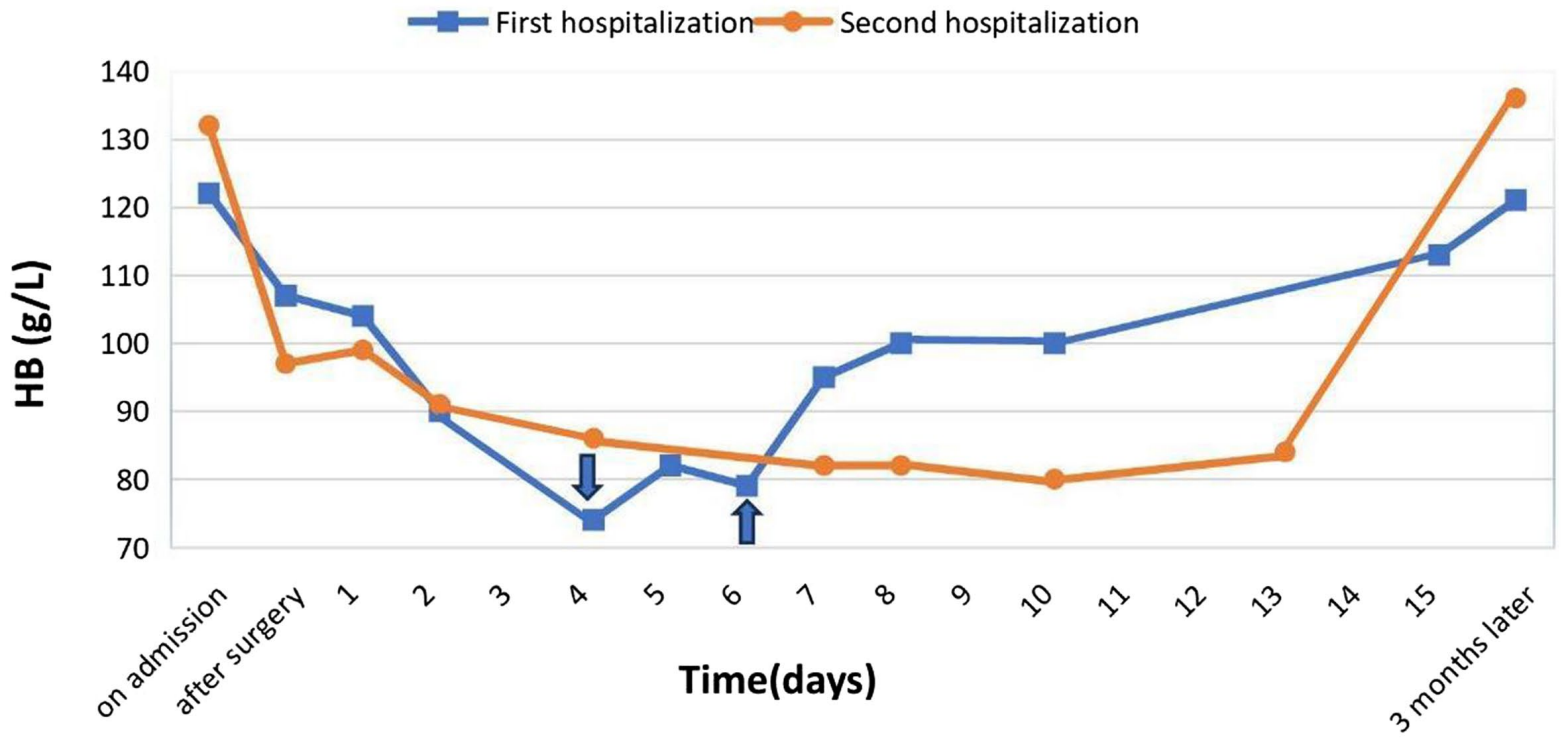
Dynamics of hemoglobin levels during two hospitalizations.

The patient’s leukocyte count was 5.38 × 10^9^/L at the beginning, but we noted an increase to 14.04 × 10^9^/L following surgery, which we assigned to the surgical procedure. After being released from the hospital, the leukocyte count returned to normal at 8.22 × 10^9^/L. Following institutional guidelines, the patient was administered cefazolin, an antibiotic of a broad spectrum, to prevent infection complications (Figure 2).

**Figure 3:**
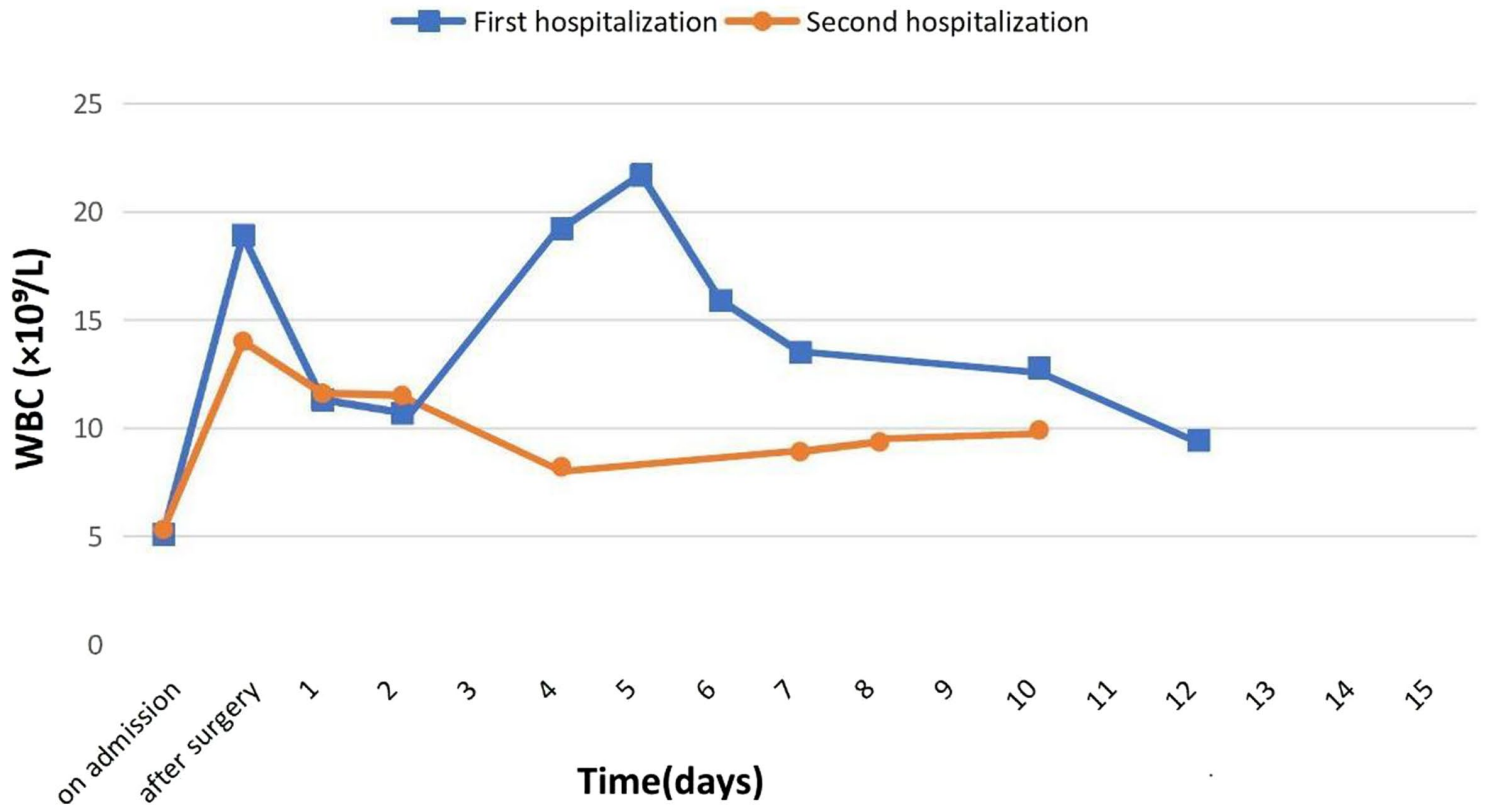
Dynamics of leukocyte levels during two hospitalizations.

Blood coagulation values and other biochemical markers, including a urine test, were within normal ranges both before and after surgery. Coagulation parameters were kept at the desired normocoagulation values following anticoagulation medication (prothrombin time: 19.2 s, prothrombin index: 0.73, INR: 1.29, fibrinogen: 2.28 g/L). Before the procedure, the patient was routinely examined for infections, such as the Wasserman reaction, HIV infection, and viral hepatitis B and C. All results were negative (Figure 3).

From the instrumental methods of investigation, an electrocardiogram and echocardiogram were performed. The results did not reveal pathologic changes. Also, an examination of the respiratory system was performed, and fluorography also did not reveal any abnormalities. During fibrogastroduodenoscopy, chronic superficial gastroduodenitis was found. The veins of the lower extremities were examined by ultrasound, the veins were passable, and no thrombosis was detected. The patient was consulted by a general practitioner and hospitalized in the orthopedic department.

According to the above, the patient was diagnosed with Crowe type IV right hip dysplasia. The total endoprosthesis of the left hip joint with Zimmer Wagner implant with shortening osteotomy from May 15, 2023. Shortening of the right lower limb by 4 cm. Mixed contracture of the right hip joint. Associated pathology included latent iron deficiency anaemia, arterial hypertension 1-degree risk 1, chronic gastroduodenitis outside the stage of exacerbation, and chronic bronchitis in remission.

### Therapeutic intervention

After informed consent was obtained and the patient’s iron deficiency was determined by assays, the patient received an infusion of ferric carboxymaltose on the first day of the preoperative period at a dose of 15–20 mg/kg of ideal body weight for anti-anemic purposes. It was diluted once intravenously over an hour using 400.0 mL of 0.9% sodium chloride solution or 1,000 mg of the drug.

In the further postoperative period, antibacterial therapy was continued with cefazolin 1,000 mg intravenously twice a day. On the first day after surgery, the patient also received postoperative analgesia in the form of narcotic analgesics: trimepiredine 20 mg intramuscularly every 6 h, and morphine hydrochloride 10 mg intravenously once a day. Further analgesia was supported by a combination of non-steroidal anti-inflammatory drugs: ketoprofen 100 mg intramuscularly twice a day for eight days, and metamizole sodium 1,000 mg intramuscularly once a day.

To prevent thromboembolic complications in the postoperative period, the patient was additionally administered low molecular weight heparin, namely nadroparin calcium 2,850 IU/0.3 mL, subcutaneously once within thirteen days after surgery. In addition, the patient took proton pump inhibitors (omeprazole 40 mg once daily) for seven days after surgery to prevent stress ulcers. There was no indication for blood transfusion. In addition, the patient received №5 mechanotherapy and kinesiotherapy as part of rehabilitation therapy.

### Surgical treatment

On the 5th day of hospitalization, the patient underwent total hip replacement of the right hip joint under general anesthesia. The duration of the operation was 110 min. Intraoperative blood loss amounted to 200 mL. To prevent blood loss we administered 1,000 mg of tranexamic acid intravenously, as well as autoreinfusion by CATS apparatus, where blood return amounted to 109 mL. Infusion therapy in the volume of 1,500 mL of 0.9% sodium chloride solution, prophylactic antibacterial therapy in the form of antibiotics of cephalosporin series of the first generation cefazolin 1,000 mg was also administered intravenously.

In the postoperative period, hemoglobin level was 97.5 g/L, on the 3rd day of the postoperative period hemoglobin was 86.0 g/L, at discharge 84.9 g/L, a decrease in hemoglobin less than 80 g/L was not observed, with the help of complex measures carried out perioperatively, we managed without a sharp decrease of hemoglobin level, also without haemotransfusion.

### Follow-up and outcomes

The patient was discharged on the 18th day of hospitalization, as she passed the 1st stage of inpatient rehabilitation. At discharge, the hemoglobin level was 84.9 g/L and the red blood cell count was 2.9 × 10^12^/L. No in-hospital haemotransfusions were performed. Intrahospital haemotransfusions were not performed due to lack of indication, also no side effects were noted.

Examination of the patient on postoperative days 28 and 90 showed that rehabilitation was successful, with no infectious complications or other adverse events. There was a high degree of compliance with these recommendations for the management of iron deficiency anaemia in the postoperative period. A week later, the patient received a repeat infusion of ferric carboxymaltose at a dose of 1,000 mg intravenously. On the 90th day, hemoglobin was 136 g/L according to the results of self-examination.

Also in the postoperative period during the first surgical intervention, there was an increase in the level of leukocytes 20–25 in the field of view in the urine, which may indicate the presence of a minor infection in the body. Such changes in the urine were not registered at the second surgical intervention.

## Discussion

The quality of life has greatly increased globally as a result of new medical advancements and innovative technologies. Previously, orthopedic surgeries were rare and had many intraoperative and postoperative complications. Currently, the number of orthopedic interventions worldwide is growing exponentially and with it, the experience and quality of orthopedic care are increasing. About 18 million orthopedic surgeries were performed in the United States alone in 2022 (5).

According to our research National Scientific Center of Traumatology and Orthopedics Named after Academician N.D. Batpenov, in 2020, 20,157 surgeries were performed in our center alone, of which 6,910 knee endoprosthetics (34.3%), 4,952 hip endoprosthetics (24.6%), arthroscopic surgeries 2,750 (13.6%) and spinal fusion 1,175 (5.8%) were performed (6). This is a significant percentage considering Kazakhstan’s modest population of 20 million. As the population ages and the proportion of overweight people increases worldwide, the number of orthopedic services is expected to increase. For instance, according to WHO, the proportion of the aging population will double by 2050, reaching 22%. Furthermore, between 1990 and 2022, the prevalence of obesity more than doubled globally (7, 8).

Each year, among all surgical procedures in the United States, primary hip and knee arthroplasty ranks in the top five most common and fastest-growing procedures (9).

Therefore, the development of strategies to improve the delivery of orthopedic care is still relevant today. In our view, before major elective joint procedures requiring significant blood loss, patients must undergo screening for iron shortage and latent anaemia, even if their hemoglobin levels are normal. Most of the present research on latent iron deficiency and anemia focuses on pregnant women, ignoring the vast majority of other people (10).

In this clinical case, the patient had normal hemoglobin values on admission to surgery in both cases, in the second case the patient was examined for concentration of iron levels, and iron deficiency was detected. Given the presence of chronic gastritis in the patient we suspected poor enteral iron absorption and therefore used an intravenous form of ferric carboxymaltose in the correction. When comparing the two hospitalizations, we noted that in the second case, the patient avoided haemotransfusions in the postoperative period.

Haemotransfusions have been widely reported to be associated with an increased risk of periprosthetic joint infection (11). Findings from the nationwide study (2025) demonstrated that allogeneic blood transfusion in the context of total knee and hip arthroplasty markedly elevated the risk of severe postoperative complications. Specifically, transfusion was associated with a higher incidence of surgical site infections (risk ratio [RR] for TKA = 17.0; for THA = 13.5), sepsis (RR for TKA = 13.4; for THA = 5.0), and pulmonary embolism (RR for TKA = 6.0; for THA = 3.5) (12). Although the risk of direct pathogen transmission through red blood cell transfusion is minimal in developed countries, transfusion-related immunomodulation may increase susceptibility to infections (13). Moreover, red blood cell transfusion is associated with a higher risk of pneumonia, surgical site infections, and other severe hospital-acquired bacterial complications, which are linked to reoperation, increased healthcare costs, and prolonged hospital stay. In turn, severe antibiotic resistance is frequently present in hospital-acquired illnesses, making treatment more difficult and raising the possibility of unfavorable results. Restrictive transfusion strategies are therefore considered an effective approach to mitigate these risks (14). However, it is important to note that some studies have demonstrated conflicting evidence, suggesting a potential protective effect of blood transfusion against PJI in specific patient populations (15).

According to the studies by Blanco et al. (16), surgical time, tourniquet application time, cement type, diabetes, obesity, ASA class, and need for blood transfusion are independently associated risk factors for periprosthetic joint infection.

In this clinical case, we observed moderate leukocyturia in a patient after the first surgical intervention. Moreover, the level of blood leukocytes in the postoperative period after the first surgery was higher compared to the level of leukocytes after the second surgery. Based on the above data, a urinary tract infection may be suspected in the patient according to a cohort study by authors Bae et al. (17), urinary tract infections were an independent risk factor for periprosthetic infections.

The uniqueness of this clinical case is that the patient was in the same conditions, but different preoperative preparation tactics were undertaken. During the first operation, the patient did not have iron deficiency correction, and iron levels were not determined. However, during the second surgery, iron deficiency was determined and corrected.

As shown in Table 1, the patient’s surgical conditions were almost identical. The duration of the operation in the first case was 10 min longer and amounted to 120 min and in the second case 110 min. The operation was performed in the first case in May and in the second case in June. According to the study, the development of infection is higher during the warm season. High temperature and low humidity favor the development of infection. According to the 2023 cohort study, late summer was an independent risk factor for periprosthetic joint infection after total joint arthroplasty. The infection rate of periprosthetic joint infection after total joint arthroplasty in late summer is higher than in other seasons (18).

**Table 1:** Comparative analysis of two hospitalizations.

Conditions	First hospitalization	Second hospitalization
Anesthesia	General anesthesia	General anesthesia
Surgeons	Same	Same
Тranexamic acid	+	+
Аutoreinfusion	140 mL	109 mL
Intraoperative infusion	1,500 mL (NaCl 0,9%)	1,500 mL (NaCl 0,9%)
Нemostatics	+ (Sodium etamsylate)	−
ICU	1 day	1 day
Length of hospitalization	22 days	19 days
Preoperative HGB	122 g/L	132 g/L
Postoperative HGB	107 g/L	97 g/L
HGB at the time of discharge	113 g/L	84 g/L
HGB after 3 months	121 g/L	136 g/L
Intraoperative blood loss	500 mL	200 mL
Postoperative blood loss (drain)	70 mL	20 mL
Operation volume	Total left hip arthroplasty with Zimmer Wagner implant with femoral shortening osteotomy.	Total hip replacement of the right hip with Zimmer Wagner implant with shortening osteotomy and derotation plate fixation
Нemotransfusion	2 doses	−
Duration of surgery	120 min	110 min
Ferric carboxymaltose 1,000 mg (Ferinject®)	−	One day before the operation
Time of year	May	June
Body mass index	24	24.8
Rehabilitation	14 days	14 days
Rehabilitation potential	low	high

The surgical site and operating team were the same. The same method of anesthesia was also used. Reinfusion was performed in both cases, in the first case 140 mL, in the second case 120 mL. But blood loss in the first surgical procedure was more and amounted to 500 mL, in the second case blood loss was less and amounted to 200 mL. Tranexamic acid and reinfusion were used in both cases. Since investigations confirm tranexamic acid lowers the incidence of infection in periprosthetic joint infection (19).

After the first operation, the patient required haemotransfusion and transfusion of 2 doses of erythrocyte-containing blood components.

Hospitalization at the first operation was longer by 3 days and was 22 and 19 days, respectively. At 3 months after surgery, control blood tests showed a higher hemoglobin level in the second case, 121 g/L and 136 g/L, respectively, even though at discharge the hemoglobin level was higher in the first case, 113 g/L and 84 g/L, respectively.

The strengths of the study are that the efficacy of iron deficiency correction was shown on one patient under equal conditions (surgery volume, anesthesia, conditions, surgical team, etc.). Also a strength of the study is the availability of medical records of patient follow-up six months after surgery during rehabilitation.

Limitations of the study in this clinical case are the lack of monitoring of iron levels, and CRP levels at the first surgery, retrospective and no scientific follow-up period at the first surgery, based on medical records data only. As well as all the limitations related to the limitations of the clinical case.

## Conclusion

In summary, this clinical case demonstrates the efficacy of iron deficiency correction in combination with complex treatment to avoid the development of severe anemia following surgery that requires a haemotransfusion. While the positive outcome in this case is encouraging, conclusions regarding the efficacy of intravenous iron infusion cannot be drawn based on a single case. The development of strategies to detect iron deficiency and its correction even at normal hemoglobin levels will be useful in preparation for major surgeries. Further studies are needed.

## Data Availability

The original contributions presented in the study are included in the article/supplementary material, further inquiries can be directed to the corresponding author/s.

## References

[1] Fowler AJ Ahmad T Phull MK Allard S Gillies MA Pearse RM. Meta-analysis of the association between preoperative anaemia and mortality after surgery. Br J Surg. (2015) 102:1314–24. doi: 10.1002/bjs.9861, PMID: 26349842

[2] Munting KE Klein AA. Optimization of pre-operative anaemia in patients before elective major surgery - why, who, when, and how? Anaesthesia. (2019) 74:49–57. doi: 10.1111/anae.14466, PMID: 30604424

[3] Wang L Liang D Huangfu H Shi X Liu S Zhong P. Iron deficiency: global trends and projections from 1990 to 2050. Nutrients. (2024) 16:3434. doi: 10.3390/nu16203434, PMID: 39458430 PMC11510637

[4] Iron Deficiency Anaemia: Assessment, Prevention, and Control. A Guide for Programme Managers. (2025). Available online at: http://www.who.int/nutrition/publications/en/ida_assessment_prevention_control.pdf (Accessed 23 February 2025).

[5] GlobalData. United States (US) Orthopedic Procedures Count by Segments and Forecast to 2030. (2025). Available online at: https://www.globaldata.com/store/report/usa-orthopedic-procedures-analysis (Accessed 20 February 2025).

[6] Bekarissov O Batpen A Ospanov K Jaxybekova G Bermagambetova G. The role of the National Scientific Center of traumatology and Orthopedics named after academician Batpenov N.D. in the formation and development of traumatology - orthopedic service of the Republic of Kazakhstan. Traumatology Orthopаedics Kazakhstan. (2021) 58:10. Special issue. doi: 10.52889/1684-9280-2021-58-4-15

[7] World Health Organization. Ageing and health. Geneva: WHO (2022).

[8] World Health Organization. Obesity and overweight. Geneva: WHO (2023).

[9] Schwartz AM Farley KX Guild GN Bradbury TL Jr. Projections and epidemiology of revision hip and knee arthroplasty in the United States to 2030. J Arthroplast. (2020) 35:S79–85. doi: 10.1016/j.arth.2020.02.030, PMID: 32151524 PMC7239745

[10] Leonard AJ Chalmers KA Collins CE Patterson AJ. A study of the effects of latent iron deficiency on measures of cognition: a pilot randomised controlled trial of iron supplementation in young women. Nutrients. (2014) 6:2419–35. doi: 10.3390/nu6062419, PMID: 24959952 PMC4073160

[11] Lucenti L Testa G Caldaci A Sammartino F Cicio C Ilardo M. Preoperative risk factors for Periprosthetic joint infection: a narrative review of the literature. Healthcare (Basel). (2024) 12:666. doi: 10.3390/healthcare12060666, PMID: 38540630 PMC10970643

[12] Maman D Nandakumar M Hirschmann MT Ofir H Haddad M Samir B. Blood transfusion in total knee arthroplasty and total hip arthroplasty: a nationwide study of complications, costs and predictive modelling. J Exp Orthop. (2025) 12:e70317. doi: 10.1002/jeo2.70317, PMID: 40655240 PMC12255935

[13] Rohde JM Dimcheff DE Blumberg N Saint S Langa KM Kuhn L. Health care-associated infection after red blood cell transfusion: a systematic review and meta-analysis. JAMA. (2014) 311:1317–26. doi: 10.1001/jama.2014.2726 Erratum in: JAMA. (2014) 312:2045. 24691607 PMC4289152

[14] Teng Z Zhu Y Liu Y Wei G Wang S Du S. Restrictive blood transfusion strategies and associated infection in orthopedic patients: a meta-analysis of 8 randomized controlled trials. Sci Rep. (2015) 5:13421. doi: 10.1038/srep13421, PMID: 26306601 PMC4549631

[15] Resende VAC Neto AC Nunes C Andrade R Espregueira-Mendes J Lopes S. Higher age, female gender, osteoarthritis and blood transfusion protect against periprosthetic joint infection in total hip or knee arthroplasties: a systematic review and meta-analysis. Knee Surg Sports Traumatol Arthrosc. (2021) 29:8–43. doi: 10.1007/s00167-018-5231-9, PMID: 30413860

[16] Blanco JF Díaz A Melchor FR da Casa C Pescador D. Risk factors for periprosthetic joint infection after total knee arthroplasty. Arch Orthop Trauma Surg. (2020) 140:239–45. doi: 10.1007/s00402-019-03304-6, PMID: 31707484

[17] Bae KJ Chae YJ Jung SJ Gong HS. Incidence and risk factors for periprosthetic joint infection: a common data model analysis. Jt Dis Relat Surg. (2022) 33:303–13. doi: 10.52312/jdrs.2022.671, PMID: 35852188 PMC9361118

[18] Yang Z Ji W Xia Y Wang X. Late summer is a risk factor for periprosthetic joint infection after total joint arthroplasty: a retrospective cohort study. Medicine. (2023) 2:e33089. doi: 10.1097/MD.0000000000033089, PMID: 36897712 PMC9997820

[19] Imanishi K Kobayashi N Kamono E Yukizawa Y Takagawa S Choe H. Tranexamic acid administration for the prevention of periprosthetic joint infection and surgical site infection: a systematic review and meta-analysis. Arch Orthop Trauma Surg. (2023) 143:6883–99. doi: 10.1007/s00402-023-04914-x, PMID: 37355487

